# Immunoexpression Pattern of Autophagy-Related Proteins in Human Congenital Anomalies of the Kidney and Urinary Tract

**DOI:** 10.3390/ijms25136829

**Published:** 2024-06-21

**Authors:** Mirko Maglica, Nela Kelam, Ilija Perutina, Anita Racetin, Azer Rizikalo, Natalija Filipović, Ivana Kuzmić Prusac, Josip Mišković, Katarina Vukojević

**Affiliations:** 1 Department of Anatomy, School of Medicine, University of Mostar, 88000 Mostar, Bosnia and Herzegovina; mirko.maglica@mef.sum.ba (M.M.); ilija.perutina@mef.sum.ba (I.P.); azer.rizikalo@mef.sum.ba (A.R.); natalija.filipovic@mefst.hr (N.F.); josip.miskovic@mef.sum.ba (J.M.); 2 Department of Anatomy, Histology and Embryology, School of Medicine, University of Split, 21000 Split, Croatia; nela.kelam@mefst.hr (N.K.); amuic@mefst.hr (A.R.); 3 Department of Pathology, University Hospital Center Split, 21000 Split, Croatia; ivanakp@mefst.hr; 4 Center for Translational Research in Biomedicine, School of Medicine, University of Split, 21000 Split, Croatia

**Keywords:** LC3B, GRP78, HSP70, LAMP2A, autophagy, nephrogenesis, congenital anomalies of the kidney and urinary tract, CAKUT

## Abstract

The purpose of this study was to evaluate the spatiotemporal immunoexpression pattern of microtubule-associated protein 1 light chain 3 beta (LC3B), glucose-regulated protein 78 (GRP78), heat shock protein 70 (HSP70), and lysosomal-associated membrane protein 2A (LAMP2A) in normal human fetal kidney development (CTRL) and kidneys affected with congenital anomalies of the kidney and urinary tract (CAKUT). Human fetal kidneys (control, horseshoe, dysplastic, duplex, and hypoplastic) from the 18th to the 38th developmental week underwent epifluorescence microscopy analysis after being stained with antibodies. Immunoreactivity was quantified in various kidney structures, and expression dynamics were examined using linear and nonlinear regression modeling. The punctate expression of LC3B was observed mainly in tubules and glomerular cells, with dysplastic kidneys displaying distinct staining patterns. In the control group’s glomeruli, LAMP2A showed a sporadic, punctate signal; in contrast to other phenotypes, duplex kidneys showed significantly stronger expression in convoluted tubules. GRP78 had a weaker expression in CAKUT kidneys, especially hypoplastic ones, while normal kidneys exhibited punctate staining of convoluted tubules and glomeruli. HSP70 staining varied among phenotypes, with dysplastic and hypoplastic kidneys exhibiting stronger staining compared to controls. Expression dynamics varied among observed autophagy markers and phenotypes, indicating their potential roles in normal and dysfunctional kidney development.

## 1. Introduction

Over the past 25 years, there has been a significant increase in the prevalence of chronic kidney diseases (CKDs) [1]. Kidney failure in children is mostly caused by CAKUT, which includes a variety of structural defects leading to kidney failure from early postnatal to late adult life [2,3]. Depending on the registry, the prevalence of these conditions is estimated to be between 4 and 60 per 10,000 births [4]. The CAKUT phenotype encompasses a range of anomalies, including an abnormal number or position of kidneys (ectopic, supernumerary, horseshoe kidney, or renal agenesis), structural defects of the renal parenchyma (hypoplasia, dysplasia, or multicystic dysplastic kidney), and congenital obstructions of the upper and lower urinary tract (ureteropelvic junction obstruction, vesicoureteral obstruction, posterior urethral valves, anterior urethral valves, or urethral atresia) [5]. There is also a range of clinical manifestations; while some children with severe CAKUT require dialysis or kidney transplantation as newborns and have significant comorbidities that impact everyday life, other children with mild CAKUT have entirely normal lives [6]. Using monogenic mouse models, we have already gathered considerable knowledge regarding the potential causes of CAKUT [7,8,9,10]. It has been demonstrated that the disruption of around 20 genes in humans, critical for nephrogenesis, may result in CAKUT, which has already been validated by different studies [11,12,13]. 

Mizushima et al. showed the important role of autophagy in renal development as a process commonly known for maintaining cellular homeostasis [14]. Our prior research demonstrated that the kidneys of *yotari* mice, whose phenotype is similar to renal hypoplasia, express autophagic markers in a different pattern than the kidneys of wild-type mice [8,15]. The importance of autophagy, especially for the cytosolic rearrangements required for proliferation and differentiation during embryogenesis and postnatal development [16,17], including podocyte maturation [18], is becoming more evident. The decrease in the podocyte differentiation marker nephrin in a study by Zhang et al. indicated that podocyte differentiation was impaired when autophagy activity was reduced. In their investigation, the Notch signaling pathway in embryonic kidneys was blocked by a γ-secretase inhibitor (DAPT), resulting in metanephros lacking podocyte precursors and proximal tubules. During the differentiation process, it was found that podocytes in the presence of DAPT had fewer autophagosomes than normal podocytes, along with decreased levels of the microtubule-associated protein 1 light chain 3 beta (LC3B) active form LC3B-II [19]. These findings imply that disruption of autophagy could set off a pathological cascade that results in CAKUT.

This study aims to explore the immunoexpression profiles of four autophagy-related proteins in both normal kidneys and kidneys affected by CAKUT. This investigation seeks to deepen our understanding of the role of autophagy in renal development and its potential implications for the pathogenesis of CAKUT.

In the proximal and distal tubules of a damaged kidney, Livingstone et al. observed increased accumulation of LC3B, a biochemical marker of autophagy [20], indicating that this protein may have an important role in the post-injury period of abnormal kidneys. The LC3 family of proteins is crucial for the extension and closure of the autophagosome double membranes. The amount of these proteins typically indicates the number of autophagosomes and autolysosomes generated, which can be correlated with the degree of autophagic activity [21]. Our recent study showed that *yotari* mice had a stronger staining intensity, mostly found in the cytoplasm of cells in the visceral layer of the Bowman’s capsule and convoluted tubule cells in embryogenic and postnatal kidneys compared to the wild type [8].

Three lysosomal-associated membrane protein 2 (LAMP2) isoforms currently known to exist are LAMP2A, LAMP2B, and LAMP2C. The distribution of these proteins, as well as their functions, differ greatly. In a chaperone-mediated autophagy (CMA) process, LAMP2A is a channel and receptor for transporting cytosolic proteins [22]. The only research that explains this receptor’s distribution in renal anomalies is that of Zhang et al. The authors found that the Ctns−/− mice have an apical distribution of LAMP2A in proximal tubule cells, and the lysosomal protein is absent in the basal areas of these cells. These observations suggest that LAMP2A trafficking is defective, which inhibits CMA activity and may cause progressive renal injury in cystinosis [23]. In our previous study [8], a similar distribution of LAMP2A was observed in *yotari* animals, as Zhang et al. reported [23], with a LAMP2A strong and diffuse signal predominantly found in the apical membranes of the convoluted tubules’ cells.

The family of conserved immunodominant proteins known as 70-kilodalton heat shock proteins (HSP70) is an essential part of the cell’s machinery for folding proteins, carrying out chaperoning tasks, and protecting cells from the harmful effects of physiological stresses. Typically, these stresses cause damage to proteins, which may lead to partial unfolding and aggregation [24]. It has been demonstrated that HSP70 inhibits nicotinamide adenine dinucleotide phosphate oxidase (NADPH oxidase), a critical stage in the pro-inflammatory cascade, thereby protecting renal epithelium [25]. Additionally, during the necrotic phase of acute tubular necrosis caused by inorganic mercury, HSP70 and HSP60 coexisted in areas of severe damage that suggests that an increased expression of HSP60 and HSP70 in HgCl2-induced acute renal failure accelerates the reconfiguration of “disordered” proteins [26].

Glucose-regulated protein 78 (GRP78) or immunoglobulin heavy chain binding protein (BiP) is a member of the heat shock protein 70 (HSP70) family [27]. Its primary role in so-called endoplasmic reticulum stress (ER stress) is to repair misfolded proteins and prevent the transit of improperly folded protein components [28]. The immunoexpression of this marker is significantly elevated in diabetic kidneys and various renal disorders, including renal fibrosis, genetic mutations of renal proteins, proteinuria, idiopathic nephrotic syndrome, and minimal change renal disease [29,30]. In our earlier research, we demonstrated that, compared to the control group of animals, the kidneys of *yotari* mice exhibit increased signal intensities in all renal structures [8].

In this study, we evaluated the spatiotemporal immunoexpression of LC3B, LAMP2A, HSP70, and GRP78 between the 18th to the 38th developmental week (dw) in human kidneys that were developing normally and kidneys that were affected by CAKUT. Using knowledge from previous gene-targeting knockout mouse studies, we wanted to observe the expression of autophagy-related proteins on these rare and valuable human kidney samples. The translation of results from earlier animal studies into human material studies might significantly affect our understanding of CAKUT and improve diagnostic and therapeutic possibilities for kidney diseases.

## 2. Results

Tubules, collecting ducts, and glomeruli of normal and CAKUT kidneys were examined for immunofluorescence staining intensity and expression patterns of LC3B, LAMP2A, GRP78, and HSP70. To quantitatively assess the immunoreactivity of these markers, the area percentage of the fetal kidney cortex in the nephrogenic zone and the juxtamedullary region was measured. The results were expressed as area percentages of the positive signal. Moreover, linear and nonlinear regression modeling examined the expression dynamics and progression of LC3B, LAMP2A, GRP78, and HSP70 over the observed developmental periods.

### 2.1. LC3B Expression

**Figure 1:**
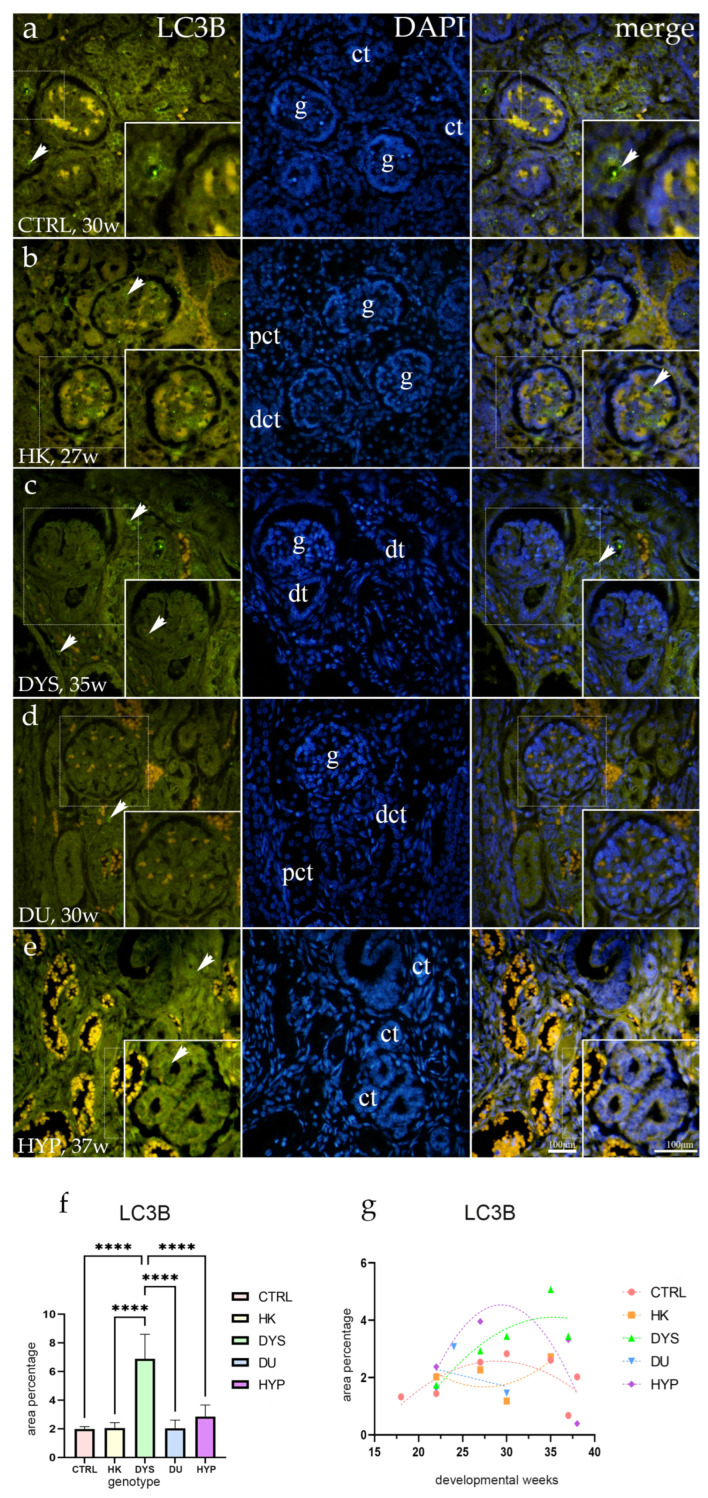
Human fetal kidney immunofluorescence staining using the microtubule-associated protein 1 light chain 3 beta (LC3B) antibody (**a**–**d**). Indicated on the 4′,6-diamidino-2-phenylindole-stained image (DAPI), the expression pattern of LC3B (green signal) is represented by arrows in the glomeruli (g), convoluted tubules (ct), proximal convoluted tubules (pct), distal convoluted tubules (dct), and dysplastic tubules (dt). Immunoexpression of LC3B, DAPI staining, and merged LC3B and DAPI in control (CTRL) at the 30th dw (**a**), horseshoe kidney (HK) at the 27th dw (**b**), dysplastic kidney (DYS) at the 35th dw (**c**), duplex kidney (DU) at the 30th dw (**d**), and hypoplastic kidney (HYP) at the 37th dw (**e**). Inserts corresponding to the dashed boxes represent the most prominent region of protein expression. At a ×40 magnification, images were captured. The scale bar is 100 μm, which refers to all images. The LC3B (**f**) area percentages in the cortex of CTRL, HK, DYS, DU, and HYP fetal kidneys are displayed as the mean ± SD (vertical line) and underwent a Tukey’s multiple comparison test after a standard one-way ANOVA test. Significant differences were indicated by **** *p* < 0.0001. Ten sample images were evaluated at each time interval. By using linear and nonlinear regression modeling of area percentages over developmental stages in the cortex of CTRL, HK, DYS, DU, and HYP fetal kidney tissues at the 18th, 22nd, 24th, 27th, 30th, 35th, 37th, and 38th dw, the expression dynamics of LC3B (**g**) were demonstrated. The mean is used to present the data.

The healthy control group’s LC3B-immunohistochemical staining was inconsistent across all observed structures. The convoluted tubules in the nephrogenic zone and the juxtamedullary region had mild punctate expression in their apical membranes. The visceral cells of developing glomeruli showed a mild, sporadic punctate signal, but parietal cells exhibited no immunoexpression (Figure 1a).

For most CAKUT phenotypes, including HK, DU, and HYP fetal kidneys, the staining pattern was similar to the control group’s, with a signal primarily seen in visceral glomerular cells (Figure 1b,d,e). Dysplastic kidneys, on the other hand, showed a distinct staining pattern, which included a sporadic signal in the visceral cells of the glomeruli, but also in the parietal cells of the Bowman’s capsule, as well as a strong, diffuse, perinuclear LC3B staining in the distal convoluted tubule cells. The mesenchymal cells surrounding convoluted tubules also showed a weak-punctate signal (Figure 1c).

Significant differences were found in the area percentage of LC3B-positive cells among the examined groups; DYS had the highest rate of LC3B-positive cells compared to CTRL, HK, DU, and HYP (*p* < 0.0001; Figure 1f).

In DYS kidneys, the proportion of LC3B-positive cells increased with time in a quadratic pattern (R^2^ = 75%). Over time, the number of LC3B-positive cells decreased in other phenotypes. Of these, only DU showed a linear trend (R^2^ = 87.9%, β = −0.15 ± 0.07), while HYP, CTRL, and HK showed negative quadratic trends (R^2^ = 50%; R^2^ = 51%; R^2^ = 63%; Figure 1g).

### 2.2. LAMP2A Expression

**Figure 2:**
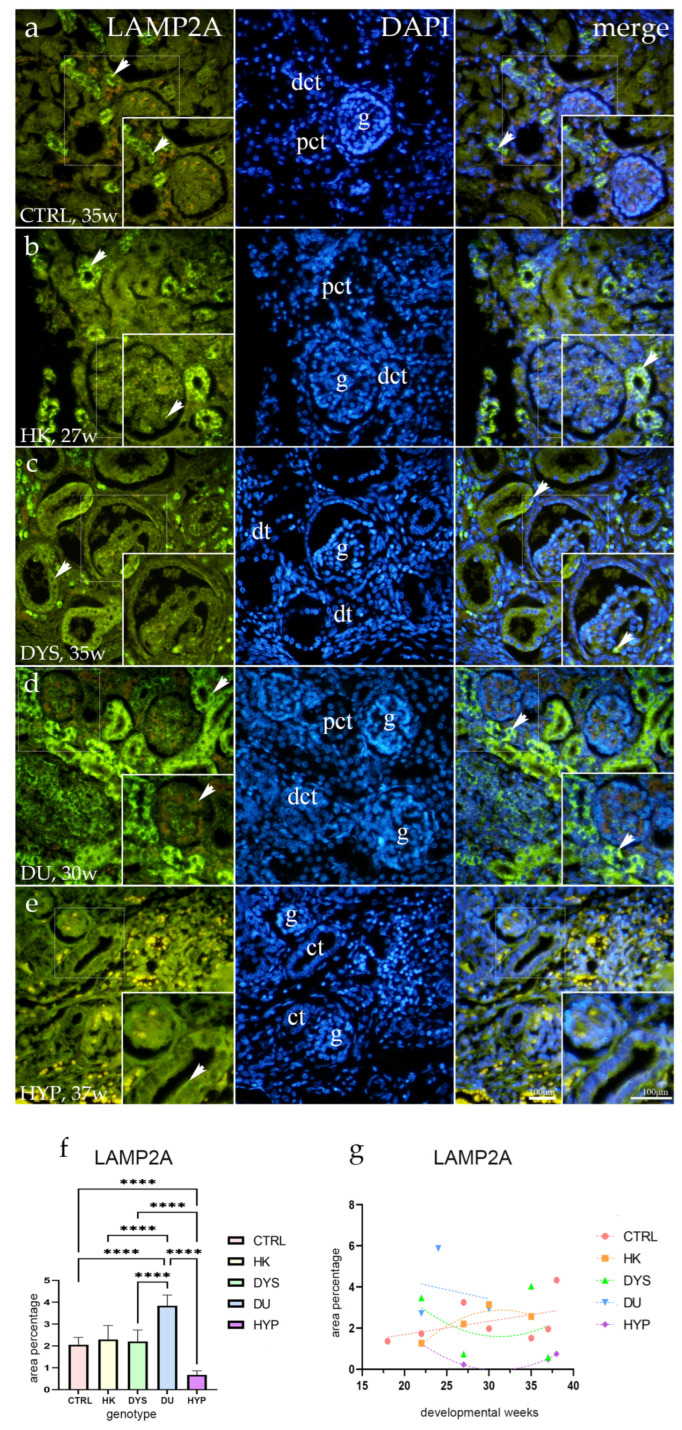
Human fetal kidney immunofluorescence staining using the lysosomal-associated membrane protein 2A (LAMP2A) antibody (**a**–**d**). Indicated on the 4′,6-diamidino-2-phenylindole-stained image (DAPI), the expression pattern of LAMP2A (green signal) is represented by arrows in the glomeruli (g), convoluted tubules (ct), proximal convoluted tubules (pct), distal convoluted tubules (dct), and dysplastic tubules (dt). Immunoexpression of LAMP2A, DAPI staining, and merged LAMP2A and DAPI in control (CTRL) at the 35th dw (**a**), horseshoe kidney (HK) at the 27th dw (**b**), dysplastic kidney (DYS) at the 35th dw (**c**), duplex kidney (DU) at the 30th dw (**d**), and hypoplastic kidney (HYP) at the 37th dw (**e**). Inserts corresponding to the dashed boxes represent the most prominent region of protein expression. At a ×40 magnification, images were captured. The scale bar is 100 μm, which refers to all images. The LAMP2A (**f**) area percentages in the cortex of CTRL, HK, DYS, DU, and HYP fetal kidneys are displayed as the mean ± SD (vertical line) and underwent a Tukey’s multiple comparison test after a standard one-way ANOVA test. Significant differences were indicated by **** *p* < 0.0001. Ten sample images were evaluated at each time interval. By using linear and nonlinear regression modeling of area percentages over developmental stages in the cortex of CTRL, HK, DYS, DU, and HYP fetal kidney tissues at the 18th, 22nd, 24th, 27th, 30th, 35th, 37th, and 38th dw, the expression dynamics of LAMP2A (**g**) were demonstrated. The mean is used to present the data.

When normal kidneys were stained with LAMP2A antibodies, the juxtamedullary region showed an intense, diffuse expression pattern in the convoluted tubules and a modest, punctate cytoplasmic signal of visceral glomerular cells. In the nephrogenic zone, PCT and DCT in nearly one-third of the examined convoluted tubules showed diffuse basolateral membranous and cytoplasmic staining, whereas the juxtamedullary region showed the occasional presence of this signal pattern (Figure 2a).

The LAMP2A immunohistochemistry pattern for HK was mostly consistent with that of the CTRL, with a punctate basolateral staining present in the majority of tubules and a diffuse signal in only a small proportion of juxtamedullary-located convoluted tubules. However, the overall intensity of the signal was decreased (Figure 2b). LAMP2A staining was equally intense in the nephrogenic and juxtamedullary regions of the DYS kidneys, with dysplastic tubules expressing a strong, punctate, and occasionally even diffuse signal both basolaterally and apically. Glomeruli exhibited weak punctate staining in the juxtaglomerular apparatus (JGA) and weak cytoplasmic staining of the visceral cells (Figure 2c). Strong, diffuse membranous and cytoplasmic staining patterns of over 80% of convoluted tubules in DU kidneys were demonstrated, accompanied by a weak punctate signal of visceral cells in glomeruli (Figure 2d). HYP models showed sporadic, weak LAMP2A immunoexpression on apical membranes of PCT (Figure 2e).

The percentage of LAMP2A-positive cells was significantly higher in CTRL when compared to HYP (*p* < 0.0001), while DU showed a significantly higher percentage score when compared to all other phenotypes (*p* < 0.0001; Figure 2f).

While the percentage of positive cells in DU kidneys gradually decreased with time (R^2^ = 95.5%, β = −0.18 ± 0.09), LAMP2A staining showed a positive linear trend in the CTRL group (R^2^ = 79.9%, β = 0.13 ± 0.064). Whereas the percentage of LAMP2A-positive cells in HK steadily increased with time, similarly following a quadratic pattern (R^2^ = 89%) with peak expression at 30 dw, DYS and HYP kidneys showed a quadratic negative trend (R^2^ = 90%; R^2^ = 99%; Figure 2g).

### 2.3. GRP78 Expression

**Figure 3:**
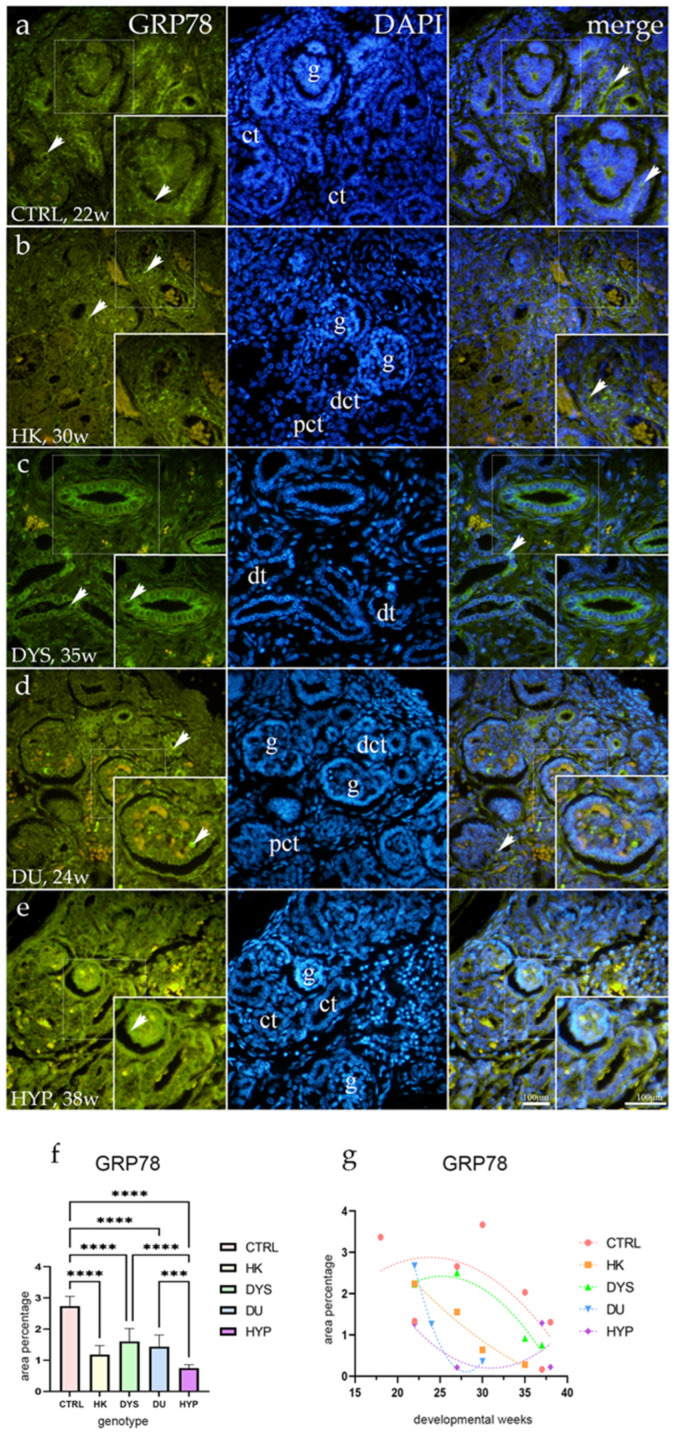
Human fetal kidney immunofluorescence staining using the glucose-regulated protein 78 (GRP78) antibody (**a**–**d**). Indicated on the 4′,6-diamidino-2-phenylindole-stained image (DAPI), the expression pattern of GRP78 (green signal) is represented by arrows in the glomeruli (g), convoluted tubules (ct), proximal convoluted tubules (pct), distal convoluted tubules (dct), and dysplastic tubules (dt). Immunoexpression of GRP78, DAPI staining, and merged GRP78 and DAPI in control (CTRL) at the 22nd dw (**a**), horseshoe kidney (HK) at the 30th dw (**b**), dysplastic kidney (DYS) at the 35th dw (**c**), duplex kidney (DU) at the 24th dw (**d**), and hypoplastic kidney (HYP) at the 38th dw (**e**). Inserts corresponding to the dashed boxes represent the most prominent region of protein expression. At a ×40 magnification, images were captured. The scale bar is 100 μm, which refers to all images. The GRP78 (**f**) area percentages in the cortex of CTRL, HK, DYS, DU, and HYP fetal kidneys are displayed as the mean ± SD (vertical line) and underwent a Tukey’s multiple comparison test after a standard one-way ANOVA test. Significant differences were indicated by *** *p* < 0.001, **** *p* < 0.0001. Ten sample images were evaluated at each time interval. By using linear and nonlinear regression modeling of area percentages over developmental stages in the cortex of CTRL, HK, DYS, DU, and HYP fetal kidney tissues at the 18th, 22nd, 24th, 27th, 30th, 35th, 37th, and 38th dw, the expression dynamics of GRP78 (**g**) were demonstrated. The mean is used to present the data.

The majority of the convoluted tubules in the control group had GRP78-positive staining. PCT showed strong, membranous, punctate staining both apically and basolaterally, whereas DCT revealed isolated diffuse staining of the apical membranes. Additionally, staining was punctate in the cells of the Bowman’s capsule’s visceral and parietal layer (Figure 3a). In both the kidneys affected by CAKUT and the healthy controls, endothelial cells of the arteries were GRP78-positive.

The CAKUT-affected kidneys have shown weaker, mostly diffuse signals of the apical membranes of convoluted tubules. Few visceral cells in glomeruli also expressed moderate punctate signal, but a substantial diffuse signal of cells in the juxtaglomerular apparatus (JGA) was seen in the DYS and DU (Figure 3b–e).

Compared to CAKUT phenotypes, the area percentage of GRP78-positive cells in controls increased significantly (*p* < 0.0001). As seen in Figure 3f, GRP78 expression was substantially lower in HYP than in DYS (*p* < 0.0001) and DU (*p* = 0.002).

In all of the examined phenotypes, the expression of GRP78 decreased during fetal development, and they all followed a quadratic trend with peak expression for control (R^2^ = 40%) at 27th, HK (R^2^ = 96%) at 22nd, DYS (R^2^ = 96%) at 27th, DU (R^2^ = 100%) at 22nd, and HYP (R^2^ = 30%) also at the 22nd developmental week (Figure 3g).

### 2.4. HSP70 Expression

**Figure 4:**
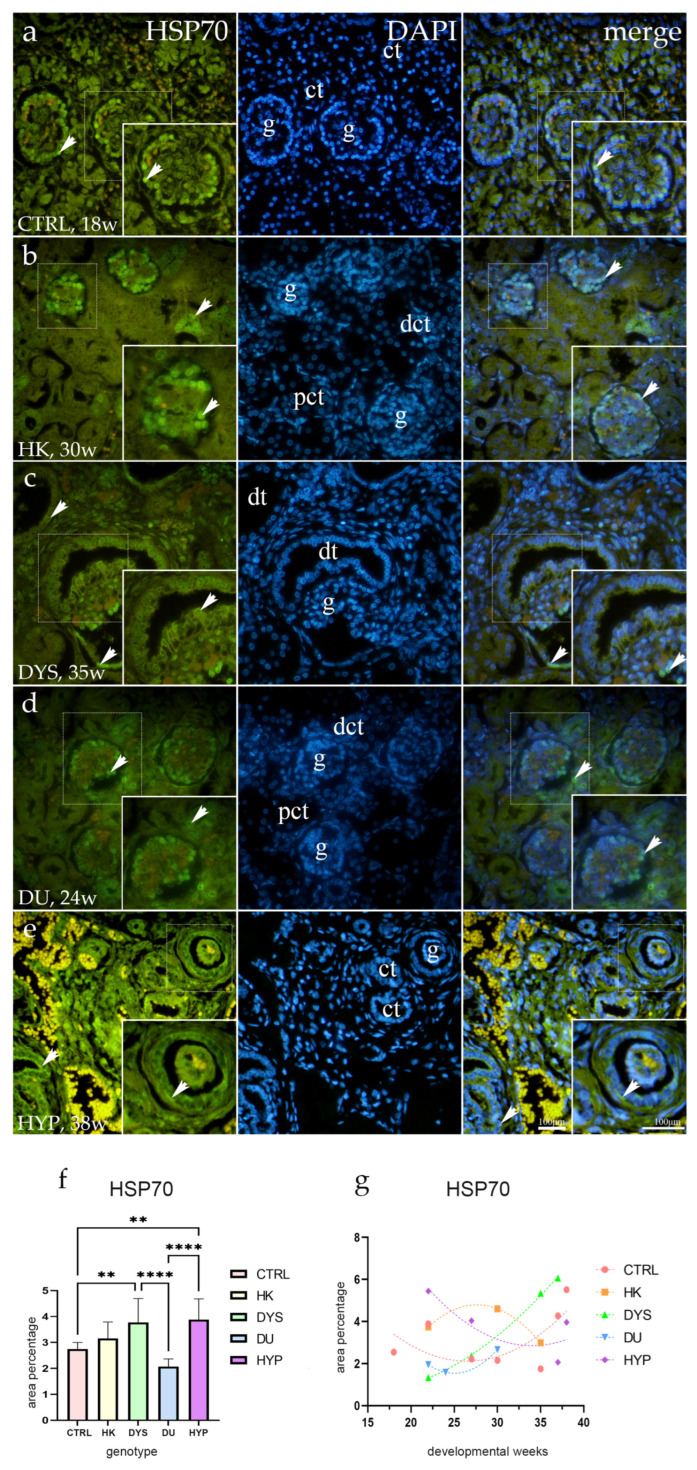
Human fetal kidney immunofluorescence staining using the heat shock protein 70 (HSP70) antibody (**a**–**d**). Indicated on the 4′,6-diamidino-2-phenylindole-stained image (DAPI), the expression pattern of HSP70 (green signal) is represented by arrows in the glomeruli (g), convoluted tubules (ct), proximal convoluted tubules (pct), distal convoluted tubules (dct), and dysplastic tubules (dt). Immunoexpression of HSP70, DAPI staining, and merged HSP70 and DAPI in control (CTRL) at the 22nd dw (**a**), horseshoe kidney (HK) at the 30th dw (**b**), dysplastic kidney (DYS) at the 35th dw (**c**), duplex kidney (DU) at the 24th dw (**d**), and hypoplastic kidney (HYP) at the 38th dw (**e**). Inserts corresponding to the dashed boxes represent the most prominent region of protein expression. At a ×40 magnification, images were captured. The scale bar is 100 μm, which refers to all images. The HSP70 (**f**) area percentages in the cortex of CTRL, HK, DYS, DU, and HYP fetal kidneys are displayed as the mean ± SD (vertical line) and underwent a Tukey’s multiple comparison test after a standard one-way ANOVA test. Significant differences were indicated by ** *p* < 0.01, **** *p* < 0.0001. Ten sample images were evaluated at each time interval. By using linear and nonlinear regression modeling of area percentages over developmental stages in the cortex of CTRL, HK, DYS, DU, and HYP fetal kidney tissues at the 18th, 22nd, 24th, 27th, 30th, 35th, 37th, and 38th dw, the expression dynamics of HSP70 (**g**) were demonstrated. The mean is used to present the data.

In the control group, HSP70 immunoexpression was observed sporadically in the visceral layer of the glomerular capsule in both the juxtamedullary and nephrogenic zones (Figure 4a). The kidneys from the DU and HK had a similar staining pattern (Figure 4b,d), but it was significantly more intense and covered nearly all of the glomerular capsule’s visceral cells. Convoluted tubules did not show any staining in these phenotypes. 

The staining pattern in the renal glomeruli of HYP kidneys was found to be quite similar. However, the apical and basolateral membranes of convoluted tubules in these specimens also showed a weak, sporadic expression of HSP70.

The dysplastic kidney expression pattern was entirely distinct from the patterns of the previously mentioned phenotypes. The Bowman’s capsule’s visceral cells still exhibited a strong, diffuse signal, with parietal cells displaying the same staining pattern. In the nuclei of epithelial cells, dysplastic tubules displayed diffuse HSP70 expression.

HYP had a considerably higher area percentage of HSP70-positive cells than CTRL (*p* = 0.0026) and DU (*p* < 0.0001). Furthermore, DYS had a substantially greater area percentage of HSP70-positive cells than CTRL (*p* = 0.0077) and DU (*p* < 0.0001; Figure 4f).

While HK and HYP showed an exponential decline with the developmental age (R^2^ = 100% and R^2^ = 71%), with peak expressions at the 27th and 22nd dw, CTRL, DYS, and DU showed a positive quadratic trend (R^2^ = 43%, R2 = 99%, and R^2^ = 100%; Figure 4g).

## 3. Discussion

Approximately 40% of all chronic kidney disease cases in children worldwide are caused by congenital anomalies of the kidney and urinary tract (CAKUT) [31]. This condition may present alone or as a component of a syndrome with extra-renal manifestations [32,33]. More than 20 single-gene abnormalities have been shown to contribute to the development of CAKUT [34]. The clinical phenotype may result from disruptions of the intricate molecular regulation at various phases of kidney development, controlled by numerous genes [35,36]. This study investigated the spatiotemporal immunoexpression of autophagy-related proteins (LC3B, LAMP2A, GRP78, and HSP70) in developing human kidneys and kidneys with congenital anomalies.

Recent studies have revealed that autophagy is crucial for kidney development and is present in several reported kidney disease models [14,37]. We highlighted the significance of autophagy in kidney development in our recent study using *yotari* mice, and we proposed that this mechanism plays an important role in averting programmed cell death [8].

Visceral cells of the glomeruli showed mostly mild LC3B staining in the control group. Convoluted tubules expressed few positive cells in the apical membranes of the kidney’s nephrogenic and juxtamedullary regions. DU, HYP, and HK also noted the same immunoexpression patterns. Since glomerular podocytes, when terminally differentiated, exhibit a high basal rate of autophagy through many vesicles in their cytoplasm resembling double-membrane autophagosomes, a positive LC3B signal in the visceral cells of the Bowman’s capsule was expected [18,38,39]. Once covalently conjugated to phosphatidylethanolamine (PE), LC3B selectively localizes to the phagophore membrane, representing the pivotal step for autophagosome formation [40]. However, dysplastic kidneys had a significantly higher percentage of LC3B-positive cells when compared to all other phenotypes. These samples showed robust, perinuclear-positive staining in the distal convoluted tubules and a sporadic signal in the parietal cells of the Bowman’s capsule and the visceral cells of the glomeruli. Furthermore, dysplastic kidneys showed a progressive increase over observed dw, whereas CTRL, HK, DU, and HYP showed a decline in LC3B immunoexpression. These findings probably suggest an important LC3B role in preventing programmed cell death, as previously described by Nakahira et al. using LC3B-deficient mice [41]. A progressive increase in oxidative stress may be the cause of the positive LC3B quadratic trend in DYS kidneys, as reported by Takashi et al. in a model of acute kidney injury (AKI) induced by the chemotherapy agent cisplatin. In this study, administering cisplatin to autophagy-deficient animals caused significant damage to the mitochondria and promoted the production of reactive oxygen species (ROS), DNA damage, and p53 activation. Thus, autophagy protects kidneys from AKI, possibly by reducing DNA damage and destroying ROS-generating mitochondria [42]. Puromycin aminonucleoside administration to rats was shown in an earlier study to cause a decrease in LC3 in podocytes and an increase in LC3 during the nephrosis recovery phase [18]. However, other studies have demonstrated the induction of glomerular ER stress following puromycin aminonucleoside administration, suggesting a connection between ER stress and autophagy in this disease model [43]. Autophagy’s substantial protective role in dysplastic kidneys is the most likely explanation for our findings since these kidneys have been shown to have abortive tubules and glomeruli primitive ducts surrounded by collars of mesenchyme, lobar disorganization, and elevated levels of oxidative stress [44,45].

GRP78, an HSP70 family stress-inducible chaperone [46], is involved in a variety of cellular functions, such as facilitating the folding and assembly of newly synthesized proteins, directing misfolded proteins toward proteasome degradation, controlling calcium homeostasis, and acting as a sensor of ER stress [47]. GRP78 likely plays a crucial role in early development due to increased ER stress during this period, where increased cell proliferation and secretion occur. GRP78’s control over essential steps, such as protein translocation into the ER lumen, mirrors its critical function observed in yeast cells. Using *Grp78* knockout mice, Luo et al. proved that the absence of this chaperone could lead to a series of pro-apoptotic pathways and early embryonic lethality. In their study, *Grp78*^−/−^ embryos showed decreased proliferation and higher apoptosis activity in addition to not growing in culture [48]. The PCT and DCT of the control group’s kidneys showed the highest immunoexpression of GRP78-positive cells in our research, indicating an important role for GRP78 during normal kidney development. The apical membranes of convoluted tubules showed weaker, mainly diffuse signals in all CAKUT phenotypes, with the HYP kidneys exhibiting a significantly smaller proportion of GRP78-positive cells. These results point to a potential protective function of GRP78 during the early stages of nephrogenesis, when its absence may cause an accumulation of unfolded or misfolded proteins, which in turn may cause a general deficiency in proliferation and an increase in the activity of apoptosis [48]. Results from previous studies indicate that certain gene mutations increase ampullary bud apoptosis and decrease ampullary bud branching, which results in a low number of nephrons and is linked to renal hypoplasia in humans [49,50,51]. This fact clarifies why GRP78 immunoexpression was noticeably lower in HYP kidneys in our study. Furthermore, we found that endothelial cells in kidneys with and without CAKUT had a strong GRP78 signal. Although this discovery has not been investigated comparably, Jin et al. demonstrated the presence of GRP78 in the vascular wall cells of striatal neurons, such as adventitial fibroblast-like cells, smooth muscle cells, and endothelial cells [52]. To better understand this finding, future research will be required to compare isolated GRP78-expression in vascular tissue between normal and CAKUT-affected kidneys. 

Examining the age-related GRP78 trend, all observed phenotypes showed a gradually declining expression, consistent with findings from earlier research indicating that GRP78 plays a major role in early embryogenesis. However, its role does not end here; research using mouse embryos at days 9.5 and 10.5 suggests that it also has a role in the later stages of embryonic development [48,53]. These results imply that GRP78 plays a critical protective role in the early development of the kidney and that its absence will result in a proliferative deficit that may cause CAKUT, namely renal hypoplasia.

We discovered that the strongest LAMP2A signal was present in cells of DU kidneys, located in convoluted tubules, and accompanied by a modest, punctate signal in the visceral cells of the glomeruli. Hypoplastic kidneys showed the weakest LAMP2A immunoexpression, mostly found on apical membranes of PCT. LAMP2A exhibited a similar pattern in our earlier investigation using *yotari* mice, with considerably higher immunoexpression in mutant specimens [8]. Since LAMP2A serves as a lysosomal receptor for CMA, variations in its rate of synthesis, control through degradation, sub-compartmentalization, and lysosomal organization all affect CMA activity [54]. Using *Lamp2a* knockout mice, Massey et al. found that cells with reduced CMA activity are more vulnerable to various stresses. This discovery may indicate that starvation-induced cell death occurs when the CMA system is completely blocked. Alternatively, compensatory activation of macroautophagy may result from mutations in the CMA pathway, allowing cells to adapt to nutritional stress without compromising their viability [55,56]. When CMA is blocked by silencing *Lamp2a*, two glycolytic enzymes, GAPDH and PGK, are transcriptionally suppressed by wild-type TP53. This decrease in glycolysis and ATP production ultimately reduces cell proliferation and increases cell death. It is possible that a malfunction in CMA activity caused by LAMP-family isoform deficiency could result in improper cell proliferation [57] and CAKUT development. Autophagy plays a crucial role in all stages of embryogenesis and the early postnatal period, as previously established in studies using various animal models. It assists in survival beyond particular developmental stages, removes organelles and protein aggregates at specific times during development, and provides a mechanism for survival when nutrients are scarce [58]. In our study, LAMP2A immunoexpression only exhibited a positive linear trend during the developmental weeks in the control group, which intriguingly demonstrated this protein’s critical role throughout the entire kidney development process. Previous research has shown that whereas LAMP2A expression decreases with age, overexpressing it during the embryonic and early postnatal stages can prevent the age-related loss in CMA, reducing damaged protein buildup and enhancing organ function [59]. Abnormal CMA receptor LAMP2A expression and localization probably occur in CAKUT kidneys, promoting oxidized protein buildup. Further research is still required to gain insights into the role of CMA in CAKUT.

The most common and well-conserved heat shock protein, HSP70, aids in the restoration of protein function following misfolding or helps it revert to its original structure. A prior study that examined the expression of HSP70 in 22 children with unilateral ureteropelvic junction obstruction (UPJO) found that the expression pattern of HSP70 was related to the length of the obstruction and was seen in the medullary collecting ducts, cortical collecting ducts, and proximal tubules [60]. A similar immunoexpression pattern was observed in our study, where dysplastic and hypoplastic tubules exhibited a strong, diffuse signal. It is believed that the functional integrity of the kidney depends on normal development and physiological cell turnover. Apoptosis modulation is essential for these mechanisms. Cumulative evidence suggests that HSP70 is critical and fundamental in modulating apoptotic pathways and oxidative stress during kidney development [61]. The study by Murer et al. suggests that vesicoureteric reflux (VUR) and the associated renal dysplasia/hypoplasia may result from a dysregulation of the complex gene network that regulates the normal development of the kidneys and urinary tract, including apoptotic pathways [62]. Given that the HSP70 signal in the kidneys from DYS and HYP patients was significantly stronger, we assume that the higher levels of oxidative stress in these specimens led to an upregulation of HSP70 expression [63]. The positive quadratic trend during dw was observed in CTRL and DYS kidneys, suggesting an important role of this chaperone in maintaining appropriate cell numbers during kidney development and cell differentiation, especially during the mesenchymal–epithelial transformation in the metanephric kidney [64].

The investigation into the spatiotemporal expression patterns of LC3B, GRP78, HSP70, and LAMP2A in both normal and CAKUT-affected kidneys sheds light on the intricate molecular mechanisms underlying kidney development and the pathogenesis of congenital anomalies. These autophagy-related proteins play pivotal roles during kidney development and in maintaining proper renal function later in life. The exact mechanisms underlying the dysregulation of these proteins in CAKUT remain to be fully elucidated. In conclusion, the intricate interplay of autophagy-related proteins in kidney development and CAKUT pathogenesis unveils novel avenues for understanding and potentially intervening in congenital renal anomalies. While oxidative stress emerges as an essential contributor to dysregulating these pathways, further research is required to unravel the molecular mechanisms underlying CAKUT. Such insights hold promise for developing innovative diagnostic and therapeutic strategies targeting CAKUT and other renal disorders, ultimately enhancing patient outcomes and quality of life.

## 4. Materials and Methods

### 4.1. Tissue Acquiring and Processing 

The University Hospital Center Split’s Department of Pathology sampled a total of 29 paraffin blocks of fetal kidney tissue (Table 1) from spontaneous miscarriages and eugenic abortions due to severe abnormalities. The blocks were processed with approval from the University Hospital Split’s Ethical and Drug Committee (class: 003-08/23-03/0015, approval number: 2181-198-03-04-23-0073) in accordance with the Helsinki Declaration. The study only included the specimens that were not macerated. The gestational age was estimated using menstruation data and an external measurement (crown–rump length) [65]. Routine histology and gross morphology were used to categorize kidney pathology.

Renal tissue was preserved in buffered formalin (4% paraformaldehyde in 0.1 M phosphate buffer saline, PBS) following the post-mortem section. The tissue was embedded in paraffin blocks, serially sliced to a thickness of 5 µm on a microtome, and mounted on slides after being dehydrated in graded ethanol solutions and washed in xylol. Hematoxylin–eosin (H&E) staining was performed on every tenth segment. The stages of normal fetal kidney development, abnormal signs in the kidneys of patients with CAKUT, and proper tissue preservation were investigated using light microscopy.

### 4.2. Immunofluorescence

Histological slides were deparaffinized in xylol, rehydrated in graded water–ethanol solutions, and then cooked in a water steamer for 30 min at 95 °C in a 0.01 M citrate buffer (pH 6.0). Following that, samples were cooled to room temperature. A protein-blocking solution (ab64226, Abcam, Cambridge, UK) was used for 20 min after washing in 0.1 M PBS to prevent nonspecific staining. After applying and incubating primary antibodies for an entire night in a humidity chamber, PBS was used as a rinse, and secondary antibodies were subsequently added and incubated for an hour (Table 2). DAPI (4′,6-diamidino-2-phenylindole) was used to visualize nuclei following rinsing in the PBS. After rinsing in the PBS, slides were coated with a coverslip and mounting material (Immuno-Mount, Thermo Shandon, Pittsburgh, PA, USA). Each primary antibody was diluted in a blocking solution at the prescribed concentration before the preadsorption test. Sections were coated with a solution containing the relevant peptide antigen. No sign of antibody staining was found. There was no evidence of nonspecific secondary antibody binding or false-positive results when primary antibodies were not included in the immunofluorescence process.

### 4.3. Data Acquisition

A Nikon DS-Ri2 camera (Nikon Corporation, Tokyo, Japan) with NIS-Elements F software version 4.60 was attached to an epifluorescence microscope (BX51, Olympus, Tokyo, Japan) to take pictures of the fetal kidney cortex (nephrogenic zone and juxtamedullary region). In 10 non-overlapping typical fields, LC3B, GRP78, HSP70, and LAMP2A were examined at ×40 magnification for a consistent exposure duration. Diffuse or punctate green staining was interpreted as positive for each autophagic marker, while yellow staining represented autofluorescence from erythrocytes or apical brush-border membranes in proximal convoluted tubules.

### 4.4. Area Percentage Image Analysis

We calculated the area percentage of the fluorescence signal in the photographs taken to quantify the immunofluorescence of the proteins under examination. The following strategies were used to process each image [66]. Initially, the “levels” tool in Adobe Photoshop, version 21.0.2 (Adobe, San Jose, CA, USA), eliminated the background signal. The green signal in the two images was then isolated by removing the red color channel using ImageJ software version 1.530 (NIH, Bethesda, MD, USA). After making duplicates of the images, one of them was given the median filter. The filtered images were subtracted from the unfiltered ones to identify the positive signal. The produced photos were thresholded using the “triangle” option and converted to 8-bit images. To ascertain the area percentage of the thresholded images, the “analyze particles” function was employed. Because there were spots without tissue in a few of the assessed images, the measured area percentage was lower than the actual area percentage. Using the magic wand tool in Adobe Photoshop, we counted the total pixels (*px*) in each image and the number of empty pixels to adjust the area percentage value. The following formula was used to determine the adjusted area percentage, which was then employed in the statistical analysis:

$$Corrected\;area\;percentage=\frac{Uncorrected\;area\;percentage\times total\;px}{total\;px-empty\;space\;px,}$$

### 4.5. Statistical Analysis

GraphPad Prism 9.0.0 (GraphPad Software, San Diego, CA, USA) was used for statistical analysis. The normal distribution was verified using the Shapiro–Wilk test. 

Every dataset pertaining to area percentage analysis was described using the F distribution, F (DFn, Dfd), where DFn denotes the degrees of freedom numerator, Dfd denotes the degrees of freedom denominator, and *p* at the probability level of *p* < 0.05 was considered statistically significant. The mean ± standard deviation (SD) was used to express the percentage of positive cells. Both linear and nonlinear regression modeling were utilized to examine the dynamics and patterns of expression for various proteins during developmental stages. In models utilized for trend description, a coefficient is displayed as a point estimate plus or minus the standard error. The coefficient of determination (R^2^) was used as a goodness of fit measure. A linear regression line’s (β) slope was used to characterize a linear trend. With GraphPad Prism 9.0.0, all graphs were made. Adobe Photoshop version 21.0.2 was used to combine the plates (Adobe, San Jose, CA, USA). For the purposes of presenting, background subtraction and contrast were applied to microphotographs.

## Figures and Tables

**Table 1:** The samples of human fetal kidneys (*n* = 29) analyzed in the study.

Developmental Weeks	Total Number of Kidney Samples	Renal Pathology
18	4	Normal kidneys (CTRL)
22	1	
27	2	
30	2	
35	2	
37	1	
38	1	
22	1	Horseshoe kidneys (HK)
30	1	
35	1	
22	2	Dysplastic kidneys (DYS)
27	1	
35	1	
37	2	
22	1	Duplex kidneys (DU)
24	1	
30	1	
22	1	Hypoplastic kidneys (HYP)
27	1	
37	1	
38	1	

**Table 2:** Antibodies used for immunofluorescence.

Antibodies		Host	Dilution	Source
Primary	Anti-LC3B/ab48394	Rabbit	1:100	Abcam (Cambridge, UK)
	Anti-GRP78/PA5-19503	Rabbit	1:300	Thermo Fisher Scientific (Waltham, MA, USA)
	Anti-HSP70/ab31010	Rabbit	1:100	Abcam (Cambridge, UK)
	Anti-LAMP2A/ab18528	Rabbit	1:100	Abcam (Cambridge, UK)
Secondary	Anti-Rabbit IgG, Alexa Fluor^®^ 488, 711-545-152	Donkey	1:300	Jackson Immuno Research Laboratories, Inc. (Baltimore, PA, USA)

## Data Availability

All data and materials are available upon request.
